# Oxidative Drugs and microRNA: New Opportunities for Cancer Prevention

**DOI:** 10.3390/cancers15010132

**Published:** 2022-12-26

**Authors:** Alberto Izzotti

**Affiliations:** 1 Department of Experimental Medicine, University of Genoa, 16132 Genoa, Italy; alberto.izzotti@unige.it; 2 IRCCS Ospedale Policlinico San Martino, 16132 Genoa, Italy

## 1. Introduction

Despite the impressive progress of therapies in recent years, cancer still remains the second leading cause of death in developed countries. Indeed, the disease remains characterised by a continuous increase in new cases (incidence). In the coming years, this ongoing cancer epidemic could overwhelm the national health systems of countries globally. This particular concern is exacerbated by cancer’s status as a chronic disease, requiring huge resources for long time periods to manage affected patients. Recently developed personalized therapies (such as small molecules and monoclonal antibodies targeting specific cancer-related pathways) are quite effective, but their high cost represents a major problem to spreading their use on a universal scale, especially in developing countries and in states devoid of effective public health systems. To face this situation, public health requires the development of new, low-cost preventive strategies with rates of high efficacy. In this regard, oxidative drugs and microRNA represent interesting new opportunities.

## 2. Oxidative Drugs

The balance between oxidant and antioxidant plays a major role in cancer development, progression, and relapses. During the carcinogenesis process, oxidative stress represents a major promoting agent. Accordingly, antioxidant intake (such as the consumption of fruit and vegetables five times a day) is a recognized strategy to decrease cancer incidence. However, the use of antioxidant drugs has obtained minimal (if not detrimental) effects in cancer therapy. This situation is related to the fact that cancer stem cells are selected, from among the global population of cancer cells, because of their ability to face oxidative damage. Standard cancer therapies are potent oxidizing agents capable of killing cancer cells. Such a mechanism is deployed via oxidizing radiation used in radiotherapy and by many chemo-therapeutic agents such as nitrosoureas (also referred as radio-mimicking drugs), anthracyclines, cisplatin, etc. These therapies kill the great majority of cancer cells but select cells able to survive because of their abundance of intracellular antioxidants, as typically occurs in the case of cancer stem cells [1]. Newly developed oxidative drugs are aimed at scavenging intracellular antioxidants from stem cancer cells, thus improving the efficacy of chemo-radiotherapies and decreasing the rate of chemo-radio resistance. Thus, oxidative therapies have been mainly used in administering oxidizing gas, such as ozone, by blood infusion. However, the effects on cancer cells in terms of cell killing and mass reduction are transient because the half-life of ozone gas in the blood is very short. This problem has been solved by binding ozone with a lipid carrier, developing a new generation of ozonized oils at high ozonides. The lipid carrier allows ozone to penetrate inside cancer cells, thus exerting its effects directly into the cytoplasm. These effects are related to the decomposition of ozone into reactive oxygen species and oxygen. Reactive oxygen species induce oxidation, thus scavenging intracellular antioxidants of cancer stem cells and oxidizing the mitochondrial membrane. This has the effect of restoring intrinsic apoptosis in cancer cells undergoing mitochondrial blockage (Warburg effect). The lipid carrier allows ozone to cross the blood–brain barrier, making it effective also towards brain cancers such as glioblastoma.

Ozone is an established anti-inflammatory agent. The inhibition of the macrophage oxidative burst exerted by ozonized oil [2] is relevant to attenuating the inflammation surrounding and penetrating cancer tissue by modulating the tumour-associated macrophages which contribute to cancer development.

Oxygen release inside solid cancers is crucial to counteract neo-angiogenesis and metastatic spreading. Indeed, hypoxia triggers (a) production and release of angiogenic factors by activating the hypoxia-inducible factor 1 [3]; (b) metastatic spreading by activating the *met* oncogene [4].

The increase in oxygen availability in normal tissue is of great relevance for cancer patients undergoing a clinical situation characterized by severe asthenia. Referred to as ‘fatigue’, the condition is caused by the muscle toxicity and bone marrow suppression induced by chemotherapies. Ozonized oil administration can increase the muscle aerobic threshold [2], improving this clinical feature in cancer patients. In this regard, ozonized oils can be considered as a physical activity-mimicking agent, exerting the physiological effects of adapted physical activity in fragile subjects. Physical activity has been demonstrated to be an effective tool to decrease the risk of cancer relapses by 40–50%, thus being reported as a ‘super-drug’ for cancer survivors [5].

The effects exerted by ozonized oils in cancer patients are summarized in Table 1. It should be noted that these effects are only obtained by using oils that have a high ozone load in terms of ozonide content [6].

Ozonized oils are composed of a lipid carrier (oleic/linoleic acid or similar) and ozone in the absence of any xenobiotic. Accordingly, their compliance is extremely high, with no significant side-effect being recorded thus far also when used at very high doses, as occurs in glioblastoma patients for example [6].

## 3. microRNA

microRNAs play a pivotal role in the carcinogenesis process and no cancer can occur in absence of a dramatic alteration of the microRNA machinery [7]. Functional consequences of mutations occurring in oncogenes are neutralized by a functioning microRNA machinery performing the control of gene expression at the epigenetic level and neutralizing messenger RNAs produced by the mutated oncogenes [8]. Only when long-term exposures to carcinogens result in an irreversible blockage of the microRNA machinery do oncogenic mutations exert their phenotypic and functional consequences, transforming normal cells into cancer cells. microRNA alterations are constantly present in cancer tissue while, by comparison, oncogenic mutations targetable by target therapies are by far less frequent, as reported by comparative studies in human lung cancer [9].

Irreversible microRNA alteration, mainly oriented towards downregulation, is a necessary condition for lung cancer development [10,11]. microRNA are short oligonucleotides that can be easily synthetized. Accordingly, a possible new strategy for cancer prevention is the administration of microRNA which aims at restoring the normal expression of the altered microRNA machinery in cancer cells. This strategy has been demonstrated to be effective in vitro, killing lung cancer cells able to survive in presence of a high load of cigarette smoke condensate [12]. However, the translatability of this approach to clinics poses some problems. First of all, the bioavailability of microRNA is difficult to obtain because microRNAs, despite their high stability, do not penetrate inside cells. Accordingly, lipid vector should be used typically, as represented by lipid nanoparticles. Used nanoparticles should be negatively charged, thus being able to bound the positively charged microRNA. There are multiple microRNA alterations in human cancer, as demonstrated for lung cancer [9], and thus the administration of single microRNA has a low probability of being effective against cancer cells. A major problem is also represented by safety. Exogenous microRNAs are recognized in the cytoplasm by specific receptors (toll-like receptors). Accordingly, a microRNA overload could result in the triggering of inflammation, lymphocyte recruitment and interferon production, as demonstrated in human patients [13]. Interferon production and lymphocyte activation could have beneficial effects, contributing to hampering cancer progression. Conversely, inflammation represents a major threat for the development of side-effects. Especially microRNA containing high percentages of guanine and cytosine in their sequence are effective in activating toll-like receptors. Each microRNA targets hundreds of genes, and thus the probability of off-target effects is quite high in the case of exogenous microRNA administration. Despite these problems, microRNA delivery has been tested as a therapeutic strategy in several clinical trials without obtaining clearcut effects thus far (www.clinicaltrials.gov, accessed on 30 November 2022). The use of microRNA delivery as an anti-cancer strategy has more recently been better defined in experimental animal models by obtaining a remarkable inhibition of lung cancer growth, using aerosolized microRNA delivery in the absence of side-effects [14]. This inhibition has been mainly attributed to a modulation in the intra-tumoral environment in favour of immune activation [14].

If many problems in need of solution still exist, limiting the clinical use of microRNA for cancer prevention and therapy, the use of these short oligonucleotides is much more validated for the early diagnosis of cancer. Indeed, microRNA are released from cancer cells into body fluids, where they can be detected by PCR and used to identify subjects at high risk of cancer development or recurrency [15].

## 4. Conclusions

Reported observations indicate that new strategies, based onto oxidative drugs and microRNA administration, are already in the advanced stages of their clinical application. Ozonized oils have been already explored for their clinical use to prevent cancer progression and relapses. However, the number of treated patients remains limited, and randomized large-scale clinical trials are required to provide adequate scientific evidence for this preventive strategy. microRNA administration poses several practical problems in clinics when these molecules are administered by intravenous injection. A possible improvement could be represented by aerosol delivery, especially for lung cancer prevention. Conversely, microRNA detection in body fluids is much nearer to readiness for clinical application. This is especially so for cancers still lacking effective screening biomarkers, such as lung cancer. However, further studies are required to establish a reproducible microRNA signature to characterise the various cancer types with.

## Figures and Tables

**Table 1:** Anti-cancer mechanisms activated by ozonized oils at high ozonides.

Body Compartment	Mechanism	Clinical Effect
Cancer cell cytoplasm	Oxidative damage	Necrosis
Stem cancer cell cytoplasm	Antioxidant scavenging	Prevention of resistance to chemo-radio therapies
Cancer cell mitochondrial membrane	Oxidative damage	Apoptosis
Cancer tissue	Decreased hypoxia	Antiangiogenic effect limiting cancer growth
Cancer tissue	Decreased hypoxia	Inhibition of metastatic spreading
Tumour-associated macrophages	Inhibition of oxidative burst and activation	Decrease in cancer associated inflammation
Skeletal muscle	Increased oxygen availability	Fatigue prevention
